# Research Progress of RAD51AP1 in Malignant Tumors of the Female Reproductive System

**DOI:** 10.1096/fj.202502048R

**Published:** 2025-10-19

**Authors:** Chengguo Zhang, Xiaoyang Liu, Zizhang Li, Jiayao Han, Jun Liang, Nan Zhou

**Affiliations:** ^1^ Graduate School of Hebei North University Zhangjiakou Hebei China; ^2^ Department of Gynecology Xingtai People's Hospital Xingtai Hebei China; ^3^ Department of Obstetrics and Gynecology Hebei Medical University Third Hospital Shijiazhuang Hebei China

## Abstract

Genomic instability may contribute to the occurrence and progression of malignant tumors of the female reproductive system. Homologous recombination repair (HRR) is vital in maintaining cellular genomic stability. RAD51‐associated protein 1 (RAD51AP1) plays a vital role in HRR, mainly participating in the formation of displacement loop (D‐loop), and is an important molecule for maintaining cellular genomic stability. Recent studies showed that RAD51AP1 was significantly overexpressed in a variety of cancer types and correlated with prognosis, suggesting that it may have a significant pro‐carcinogenic effect. However, the mechanism underlying its pro‐carcinogenic effect remains unclear, which may be closely associated with cancer stemness. Meanwhile, RAD51AP1 also plays an important role in resistance to radiotherapy and chemotherapy. Exploring RAD51AP1 and its regulatory molecules may provide new targets for overcoming cancer progression and treatment resistance. Here, we reviewed the latest research on RAD51AP1 in female reproductive system tumors and summarized its differential expression and prognostic implications. In this review, we also outlined the potential mechanisms of its procancer and drug resistance‐promoting effects to provide several potential directions for further research.

## Graphical abstract

RAD51AP1 is highly expressed in malignant tumors of the female reproductive system, exerting a pro‐carcinogenic effect and showing a close association with poor prognosis. High expression of RAD51AP1 can promote tumor cell proliferation, invasion, migration, and stemness maintenance, inhibit apoptosis, and mediate chemoresistance as well as radioresistance.

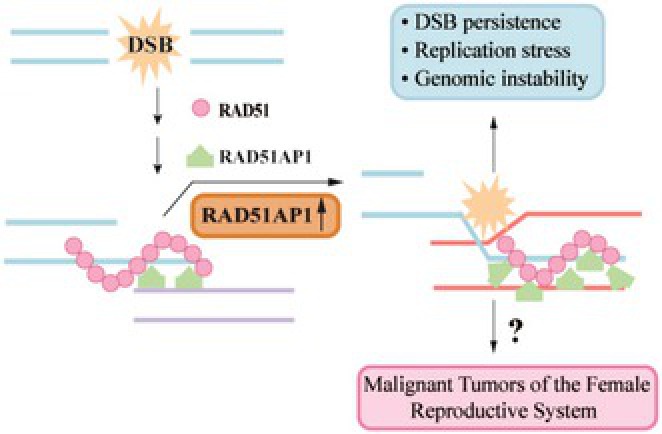

## 1 Introduction

Malignant tumors of the female reproductive system represent a significant global health threat to women [1]. Among these, ovarian cancer exhibits the highest mortality rate [2]. While the widespread use of Human Papillomavirus (HPV) vaccines has led to a decrease in cervical cancer incidence, treatment options for patients with advanced or recurrent disease remain limited [3]. Endometrial cancer, strongly associated with metabolic disorders, is increasingly being diagnosed in younger patients [4]. In contrast, breast cancer ranks as the most prevalent malignant tumor in women worldwide, with incidence rates continuing to rise [5]. Despite advancements in surgical techniques, targeted therapies, and chemotherapy regimens, challenges such as drug resistance, recurrence, and tumor heterogeneity continue to impede improvements in patient prognosis.

Emerging evidence identifies genomic instability as a key factor in tumorigenesis, with the dysregulation of DNA damage repair (DDR) pathways playing a critical role [6, 7, 8, 9]. Among DDR mechanisms, homologous recombination repair (HRR)—a high‐fidelity pathway for repairing DNA double‐strand breaks (DSB)—has been closely linked to cancer susceptibility and treatment response [10, 11]. Dysfunctional HRR not only elevates cancer risk but also influences sensitivity to chemotherapy and poly‐ADP‐ribose polymerase (PARP) inhibitors [12, 13, 14].

RAD51‐associated protein 1 (RAD51AP1), a key cofactor in HRR, interacts with RAD51 to promote homologous DNA strand pairing and exchange, thereby maintaining genomic stability [15]. RAD51 recombinase forms a nucleoprotein filament on resected single‐stranded DNA (ssDNA) at the damage site. The RAD51‐ssDNA nucleoprotein filament, also called the presynaptic filament, captures duplex DNA and generates a D‐loop upon locating a homologous DNA target [16]. RAD51AP1 can recruit RAD51 and promote the binding of RAD51 to the single‐end invasion intermediate (SEI) DNA, thereby stimulating the formation of D‐loops [17]. Recent studies have highlighted the aberrant overexpression of RAD51AP1 in various solid tumors, including breast, prostate, and colorectal cancers [18, 19]. This dysregulation is thought to drive tumor proliferation, metastasis, and therapy resistance by modulating DNA repair efficiency, cell cycle progression, and cancer stem cell properties [20].

However, the biological functions and clinical significance of RAD51AP1 in female reproductive system malignancies remain insufficiently explored. This review synthesizes current research on the molecular mechanisms of RAD51AP1 in these tumors, incorporating both preclinical and clinical data to clarify its roles in tumor initiation, treatment resistance, and prognosis. Additionally, potential therapeutic strategies targeting RAD51AP1 are discussed, offering new insights into overcoming drug resistance and guiding future translational research in female reproductive system cancers.

## 2 Overview of RAD51AP1

### 2.1 Biological Characteristics

RAD51AP1, a vertebrate‐specific DNA repair regulator, is highly conserved across evolutionary lines. First identified in 1997 by Mizuta et al. through yeast two‐hybrid screening, it was initially named Pir51 (human) and RAB22 (murine) [21]. The gene is located on the short arm of human chromosome 12 (12p13.1), spanning 12 exons and encoding a 352‐amino acid nuclear protein [22].

**FIGURE 1:**
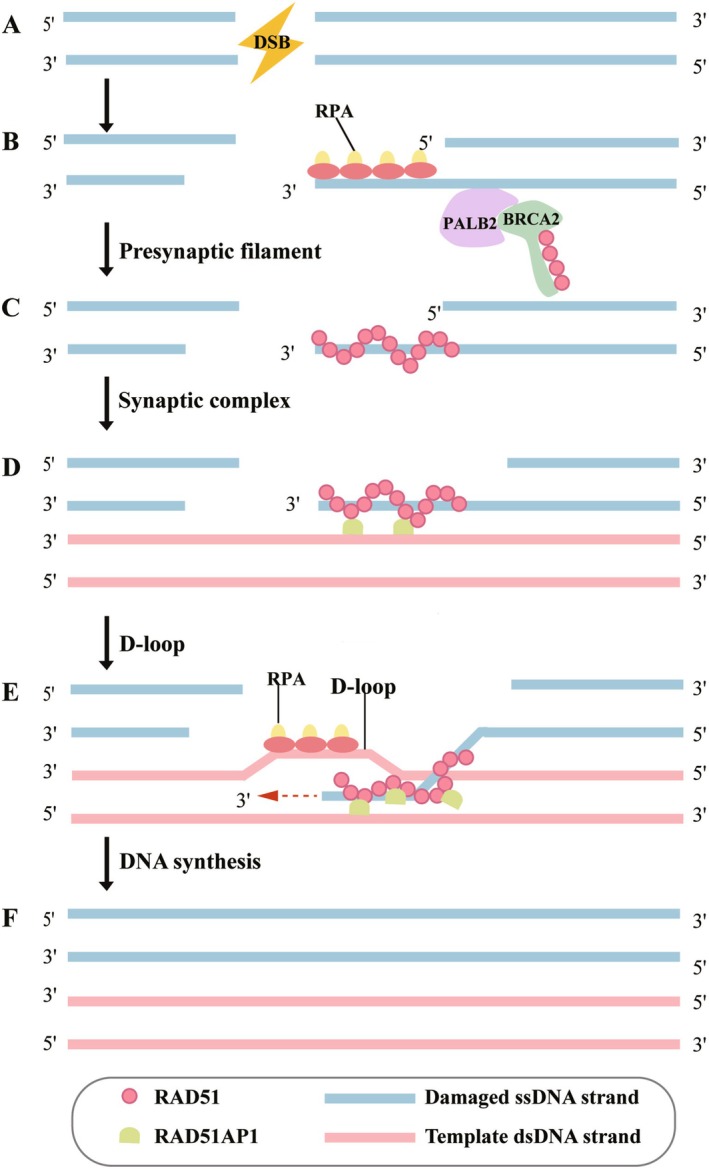
The role and function of RAD51AP1 in HRR. (A) DSB. (B) Resected RPA‐coated 3'ss DNA overhang. (C) BRCA2 interacts with PALB2 to recruit RAD51, which displaces RPA, allowing RAD51 to bind to ssDNA. (D) RAD51AP1 specifically recognizes RAD51 and stimulates RAD51 activity. (E) RAD51AP1 is primarily involved in synaptic complex formation and strand invasion, and stimulates the formation of D‐loop. (F) HRR is completed.

Structural studies reveal the modular architecture of RAD51AP1 (Figure 1): The N‐terminal domain (1–80 aa) contains a double‐helix bundle that specifically binds to the RAD51 ATPase domain, stabilizing nucleoprotein filaments [23]; the central disordered region (81–200 aa) includes dynamic phosphorylation sites that mediate interactions with repair factors, such as breast cancer susceptibility gene 2 (*BRCA2*) and partner and localizer of *BRCA2* (*PALB2*) [24, 25, 26]; the C‐terminal domain (201–352 aa) features dual zinc finger motifs that interact with the KU70/80 complex, facilitating microhomology‐mediated pairing [23, 27, 28]. Cryo‐electron microscopy (cryo‐EM) studies further confirm [25, 29] RAD51AP1's role as a molecular scaffold, promoting the formation of RAD51 nucleofilaments into right‐handed helical superstructures and stabilizing D‐loops.

RAD51AP1 orchestrates key steps in HRR by forming complexes with Replication Protein A (RPA) and RAD51, including homologous pairing and strand transfer [30]. Mechanistically, RAD51AP1 regulates HRR through a three‐phase model: (1) Damage recognition: The N‐terminal domain binds RAD51‐RPA filaments, preventing premature cleavage by meiotic recombination 11 (MRE11) nuclease [31, 32]; (2) Strand invasion: The C‐terminal domain induces donor DNA bending, lowering energy barriers for homologous pairing [23, 25]; (3) Synthesis extension: RAD51AP1 recruits proliferating cell nuclear antigen (PCNA) and DNA polymerase η (Polη) to facilitate repair synthesis [25, 33].

### 2.2 Pathological Mechanisms

RAD51AP1's regulatory network is critical for maintaining genomic stability. In somatic cells, its expression is regulated bidirectionally by the E2F transcription factor 1 (*E2F1*), which promotes its expression, and hsa‐miR‐506‐3p, which inhibits it [34, 35]. Upon DNA damage, DSBs activate DDR pathways that are dependent on ataxia telangiectasia‐mutated gene (*ATM*) and ataxia telangiectasia and Rad3‐related protein (ATR). Following DSB detection, *ATM* phosphorylates *E2F1*, relieving the inhibition of Rb protein [36, 37, 38] and subsequently upregulating *RAD51AP1* transcription [34, 39]. ATR activation requires RPA‐coated ssDNA [40], and ATR then phosphorylates the S120 site of RAD51AP1 (located in its DNA‐binding domain), enhancing its interaction with DNA/RAD51 and potentially improving HRR efficiency [41, 42]. Disruption of this intricate regulatory mechanism, particularly through tumor‐suppressive microRNAs (miRNAs) such as hsa‐miR‐506‐3p targeting the 3′ untranslated regions (3'UTRs) of HRR pathway genes, reduces HRR fidelity, leading to the “BRCAness” phenotype [43, 44].

Clinical studies have demonstrated significant overexpression of RAD51AP1 in tissues and metastases of various malignant tumors, with high expression correlating with poor prognosis [45]. These findings suggest a close involvement of RAD51AP1 in tumor initiation and progression. RAD51AP1 may also promote tumorigenesis and progression through other mechanisms, which can stimulate the alternative lengthening of telomeres (ALT) to maintain the telomere length of tumor cells, thereby promoting the proliferation of tumor cells [25]. Current studies have revealed that in ALT‐positive cancer cells, RAD51AP1 stabilizes itself through MMS21‐mediated SUMOylation, and recruits POLD3/RAD52 proteins to initiate break‐induced telomere DNA synthesis (BITS), thereby maintaining telomere homeostasis and promoting cell proliferation [46]. A study by Yadav et al. [47] indicates that RAD51AP1 can also promote telomere elongation and thereby facilitate tumor cell proliferation in a RAD52‐independent manner by binding to TERRA to form R‐loops and stabilize G‐quadruplexes (G4s) structures. Additionally, RAD51AP1 has been implicated in promoting resistance to radiotherapy and chemotherapy. Its extensive role in tumor initiation, progression, and drug resistance positions RAD51AP1 as a promising target for tumor inhibition and overcoming drug resistance.

## 3 RAD51AP1 and Malignant Tumors of the Female Reproductive System

### 3.1 RAD51AP1 and Ovarian Cancer

Ovarian cancer, the leading cause of mortality among gynecological malignancies, exhibits significant clinical heterogeneity [48]. According to 2024 data from the National Cancer Center of China [49], the global age‐standardized incidence rate of ovarian cancer is 6.6 per 100,000, with a five‐year survival rate below 45%. Approximately 70% of patients are diagnosed at stages III/IV [50, 51, 52]. This dual challenge in diagnosis and treatment highlights the importance of overcoming clinical bottlenecks by elucidating the mechanisms through which key regulatory factors in DDR, such as RAD51AP1, contribute to the onset, progression, and therapeutic resistance of ovarian cancer.

Bioinformatics and experimental studies have confirmed that RAD51AP1 facilitates ovarian cancer progression through the synergistic action of multiple pathways. Filipe et al. [53] conducted a bioinformatics analysis of 419 primary ovarian cancer cases, 8 recurrent ovarian cancer cases, and 88 normal ovarian tissue samples from The Cancer Genome Atlas (TCGA)—Ovarian serous cystadenocarcinoma (OV) databases and Genotype‐Tissue Expression (GTEx) consortia project (gtexportal.org). They observed that RAD51AP1 expression in ovarian cancer tissues, particularly in primary and recurrent foci, was significantly higher than in normal tissues. This finding was further validated by subsequent immunohistochemical (IHC) analyses, which revealed that RAD51AP1 overexpression correlated with the downregulation of tumor‐suppressive miRNAs, such as hsa‐miR‐215‐5p, hsa‐miR‐193b‐3p, and hsa‐miR‐140‐3p [54, 55].

The critical role of RAD51AP1 in mediating various ovarian cancer cell phenotypes, including proliferation, invasion, migration, and apoptosis inhibition, has been confirmed through in vitro experiments. Chudasama et al. [56] transfected SKOV3 ovarian cancer cells with si‐RAD51AP1 and observed a significant reduction in cell proliferation. Additionally, RAD51AP1 knockdown led to decreased expression of the stemness‐related protein sex‐determining region Y‐box2 (SOX2), metastasis‐promoting protein SNAI1, and the mammalian target of rapamycin (mTOR) signaling pathway, suggesting that RAD51AP1 may regulate cell proliferation via mTOR signaling. Another study found that downregulation of RAD51AP1 inhibited the proliferation, migration, and invasion of ovarian cancer SKOV3 and HEY cells [57]. Transcription levels of SMAD2‐4 and TGFBR1 were significantly reduced, along with a decrease in p‐SMAD2 and p‐SMAD3 expression, indicating that RAD51AP1 may promote tumor proliferation through the transforming growth factor‐β (TGF‐β)/Smad signaling pathway. Furthermore, the same research confirmed that high RAD51AP1 expression was linked to an immunosuppressive tumor microenvironment, characterized by Th2 cell accumulation and CD8^+^T cell inhibition, accelerating tumor progression. Meanwhile, Lin et al. [58] identified RAD51AP1 as significantly upregulated in ovarian cancer tissues through bioinformatics analysis (ovarian cancer datasets GSE18520, GSE40595, and GSE36668 were downloaded from the Gene Expression Omnibus (GEO) database), suggesting its oncogenic potential. In vitro experiments demonstrated that RAD51AP1 is highly expressed in OVCAR3 ovarian cancer cells. Upon RAD51AP1 knockdown, the cyclin‐dependent kinase 1 (CDK1)/phosphatidylinositol 3‐kinase (PI3K)/protein kinase B (AKT) pathway was inhibited, leading to cell cycle arrest in the G0/G1 phase and apoptosis.

High RAD51AP1 expression is often associated with poor prognosis. Sankaranarayanan et al. [59] utilized the Tensor Generalized Singular Value Decomposition (Tensor GSVD) method to analyze data from 249 patients with ovarian serous cystadenocarcinoma in the TCGA‐OV database. Their findings revealed that patients with high RAD51AP1 expression had significantly lower overall survival (OS) compared to those with low expression. Similarly, Chudasama et al. [56] analyzed tissue and blood samples from patients, and their computer simulation results indicated that high RAD51AP1 expression correlated with shorter OS. Zhao et al. [57] conducted bioinformatics analysis and found that ovarian cancer patients with high RAD51AP1 expression had poor OS and progression‐free survival (PFS). Lin et al. [58] confirmed through the same method that RAD51AP1 expression in ovarian cancer was negatively correlated with OS.

High expression of RAD51AP1 can also contribute to tumor resistance to chemotherapy, although the underlying mechanisms remain unclear. Zeng et al. [60] conducted in vitro overexpression and knockdown experiments using a CD133‐positive (CD133+) OVCAR4 cell model. They found that overexpressing RAD51AP1 not only significantly promoted the proliferation and self‐renewal of CD133+ OVCAR4 cells but also increased the expression levels of stemness markers, including octamer‐binding transcription factor 4 (OCT4), NANOG, Kruppel‐like factor 4 (KLF4), and SOX2. Additionally, RAD51AP1 overexpression enhanced cell tolerance to chemotherapeutic drugs. In contrast, knocking down RAD51AP1 significantly inhibited cell proliferation and self‐renewal, resensitizing the cells to chemotherapy. This suggests that drug resistance may be linked to the stemness maintenance effect mediated by RAD51AP1. Furthermore, high RAD51AP1 expression may contribute to resistance to platinum‐based chemotherapy by enhancing the DNA repair capacity of ovarian cancer cells, with its expression being negatively regulated by hsa‐miR‐140‐3p [55]. High‐dose selenides have also been shown to synergize with cytotoxic chemotherapy drugs, partially reversing drug resistance [61]. Song et al. [62] reported that in ovarian cancer OVCAR4 cells, carboplatin significantly upregulated RAD51AP1 expression, while combining carboplatin with selenous acid led to a significant reduction in RAD51AP1 levels. This suggests that selenides may reverse carboplatin resistance by inhibiting RAD51AP1 expression. Based on these findings, relevant phase I clinical trials have been initiated.

Collectively, existing evidence [55, 56, 57, 58, 59, 60, 61] indicates that RAD51AP1 is abnormally overexpressed in ovarian cancer tissues and correlates significantly with poor prognosis. Its mechanism of promoting tumor initiation and progression through a network of multiple signaling pathways has been experimentally validated. As a key regulatory factor in the HRR pathway, RAD51AP1 mediates chemoresistance by enhancing DNA repair and maintaining stem cell properties. Targeted inhibition of RAD51AP1 effectively reverses drug resistance, particularly by improving the sensitivity of ovarian cancer cells to platinum‐based therapies [55, 60, 62]. Therefore, in‐depth studies on RAD51AP1 not only help elucidate the molecular pathological processes of ovarian cancer but also provide a theoretical basis for developing novel targeted therapies, with significant clinical translational potential. However, current research has primarily focused on ovarian serous carcinoma, and its role in other subtypes, such as mucinous or endometrioid carcinoma, needs to be verified using multi‐center clinical samples and cross‐subtype in vitro and in vivo models. Furthermore, previous studies have only analyzed the expression correlation between RAD51AP1 and key signaling pathway proteins, without investigating their direct molecular interactions. Future research should focus on deciphering the direct interaction network between RAD51AP1 and key pathway proteins, developing specific inhibitors to block its oncogenic signaling, and establishing personalized treatment strategies based on RAD51AP1's functional status.

### 3.2 RAD51AP1 and Cervical Cancer

Cervical cancer is the fourth leading cause of cancer‐related deaths among women worldwide [63], with its pathogenesis primarily driven by persistent infection with high‐risk HPV (HR‐HPV). Studies have shown that during the malignant transformation of cervical cancer, key genes involved in DNA repair, cell proliferation, growth factor activation, and angiogenesis are abnormally upregulated. This genomic instability accelerates the progression of HPV‐infected cells to invasive carcinoma [64]. Despite the widespread use of HPV vaccines and early screening, which have significantly reduced the incidence rate, the five‐year survival rate for patients with advanced disease remains under 50%. Moreover, the proportion of younger patients is steadily increasing, and treatment resistance is becoming a more prominent challenge [3, 65, 66]. As such, further exploration of novel molecular regulatory mechanisms underlying cervical carcinogenesis is urgently needed to improve clinical outcomes.

Recent studies have focused on the role of DNA repair proteins, particularly RAD51 family members, in cervical cancer. Previous research has found that RAD51 is overexpressed in cervical squamous cell carcinoma, correlating with enhanced proliferation, migration, and invasion [67]. This upregulation likely contributes to resistance to chemoradiotherapy by promoting DNA repair [41, 68]. Wu [69] analyzed mRNA expression profiles from 304 cervical cancer cases and 3 normal cervical controls using the TCGA‐Cervical squamous cell carcinoma and endocervical adenocarcinoma (TCGA‐CESC) database. The analysis revealed that *RAD51AP1* mRNA was upregulated in cervical cancer tissues. As a new inducible gene of the important transcriptional activator *E2F1*, RAD51AP1 influences tumor initiation and progression and plays roles in cell cycle progression, proliferation, apoptosis, and differentiation. These findings suggest that RAD51AP1 may act as an oncogene in cervical cancer development, although its expression does not show a significant correlation with prognosis. Liang et al. [70] first examined RAD51AP1 expression in cervical cancer tissues and adjacent non‐cancerous tissues using IHC. They found significantly higher RAD51AP1 expression in cervical cancer tissues compared to adjacent tissues and also observed high expression in cervical cancer SiHa cells. In subsequent in vitro experiments, transfection of SiHa cells with si‐RAD51AP1 significantly reduced cell proliferation and colony‐forming ability, while apoptosis increased markedly. These results suggest that inhibiting RAD51AP1 expression can suppress cell proliferation and promote apoptosis in cervical cancer cells. Further analysis revealed that knocking down RAD51AP1 led to a significant reduction in the expression of TGF‐β1, p‐SMAD2, and p‐SMAD3 proteins, confirming that inhibiting RAD51AP1 suppresses the TGF‐β/Smad pathway, inhibits proliferation, and promotes apoptosis. These findings indicate that targeting RAD51AP1 may provide a novel therapeutic strategy for cervical cancer treatment.

High expression of RAD51AP1 in cervical cancer has also been shown to promote resistance to chemotherapy and radiotherapy. Studies have demonstrated that knocking down RAD51AP1 in HeLa cells significantly increases their sensitivity to mitomycin C (MMC) [30]. Ni et al. [71] confirmed that in irradiated cervical cancer SiHa and ME‐180 cells, RAD51AP1 expression levels increased significantly with escalating radiation doses. Notably, JQ1, an inhibitor of the transcriptional regulator bromodomain‐containing protein 4 (BRD4), effectively inhibits RAD51AP1 transcription, thereby enhancing the radiosensitivity of tumor cells. The underlying molecular mechanism involves BRD4 binding directly to the promoter region 350 bp upstream of the RAD51AP1 gene to activate its expression. JQ1 blocks BRD4 function, leading to downregulation of RAD51AP1 transcription.

These findings indicate that RAD51AP1 not only contributes to the initiation and progression of cervical cancer but also holds potential as a radiosensitizer. By regulating DDR and key signaling pathways, it influences tumor cell proliferation, apoptosis, and therapeutic resistance, making it a highly promising therapeutic target. Targeting RAD51AP1 could offer new theoretical foundations and clinical strategies for precision treatment in cervical cancer. However, several aspects of RAD51AP1's role in cervical cancer remain unresolved: (1) Most studies are based on a single cell line, and its applicability to other cervical cancer cell lines or clinical samples needs further verification; (2) While its association with radiosensitivity is suggested, the role and molecular mechanisms of RAD51AP1 in chemotherapy resistance still require deeper investigation; (3) As a potential therapeutic target, the development and preclinical validation of specific RAD51AP1 inhibitors are still in the early stages. Advancing research in this area will facilitate the translation of basic findings on RAD51AP1 into clinical applications.

### 3.3 RAD51AP1 and Endometrial Cancer

Endometrial carcinoma refers to a group of malignant tumors originating from the glandular epithelium of the endometrium [72]. Its global incidence has been steadily increasing, with the age‐standardized incidence rate of endometrial carcinoma reaching 8.4 per 100,000 population, highlighting the significant threat it poses to women's health [73]. Early‐stage diagnosis is possible in 80% of endometrial carcinoma patients, with tumors confined to the uterus, and these patients have a five‐year survival rate exceeding 95%. However, 15% to 20% of patients experience recurrence or metastasis. In cases of local dissemination or distant metastasis, the five‐year survival rates drop to 68% and 17%, respectively [74]. Despite advancements in precision treatment guided by molecular typing, significant challenges remain. Patients with advanced or recurrent endometrial carcinoma have an extremely poor prognosis, and the application scope of targeted therapies is still limited, driving the search for new molecular targets [75].

RAD51AP1, a key regulator of DNA repair and metabolic reprogramming, has recently emerged as a significant factor in endometrial carcinoma research. Liu et al. [20] conducted a bioinformatics analysis across various cancer types and found that RAD51AP1 is highly expressed in endometrial carcinoma tissues, with expression levels positively correlating with the mRNA Stemness Index (mRNAsi), a marker of stemness. This suggests that RAD51AP1 may promote the initiation and progression of EC by enhancing tumor stemness. Wang et al. [76] reported that overexpression of hsa‐miR‐383‐5p significantly reduces RAD51AP1 expression. Their study showed that hsa‐miR‐383‐5p inhibits RAD51AP1 expression by directly targeting its 3'UTR, indicating RAD51AP1's involvement in regulating preadipocyte proliferation and differentiation, which may link it to the pathogenesis of obesity‐related EC. Huang et al. [77] analyzed mRNA expression data from TCGA‐Uterine Corpus Endometrial Carcinoma (TCGA‐UCEC) and found that RAD51AP1 expression is significantly elevated in EC tissues. Although no direct correlation with prognosis was found, patients with high RAD51AP1 expression had significantly lower survival rates. In vitro experiments confirmed that RAD51AP1 expression is elevated in various endometrial carcinoma cell lines (RL95‐2, Ishikawa, HEC‐1‐A, HEC‐1B). Overexpression of RAD51AP1 promoted the proliferation of endometrial carcinoma cells, while knockdown of RAD51AP1 significantly inhibited cell viability, suggesting its involvement in the malignant transformation of endometrial carcinoma. Further studies revealed that downregulation of RAD51AP1 significantly reduced the median inhibition concentration (IC50) of 5‐fluorouracil (5‐FU), while its upregulation substantially increased the 5‐FU IC50, indicating that RAD51AP1 expression is closely linked to endometrial carcinoma cell sensitivity to 5‐FU. Molecular mechanism studies demonstrated that RAD51AP1, activated by *E2F7*, inhibits endometrial carcinoma cell sensitivity to 5‐FU through the fatty acid metabolism pathway.

Although existing studies have preliminarily established the critical role of RAD51AP1 in the initiation and progression of endometrial carcinoma, current conclusions primarily rely on bioinformatics analyses and in vitro experiments, lacking validation in PDX or cell‐line derived xenograft (CDX) models. Additionally, the specific regulatory mechanisms driving endometrial carcinoma progression require further investigation: (1) The differential expression of RAD51AP1 across various molecular subtypes of endometrial carcinoma has yet to be comprehensively defined; (2) The interaction between RAD51AP1 and key signaling pathways in endometrial carcinoma, such as PI3K/AKT and Wnt/β‐catenin, remains unclear; (3) The role of RAD51AP1 in the cascade of obesity‐related carcinogenesis through preadipocyte differentiation and its potential cross‐regulation with the estrogen signaling pathway remains to be elucidated. As research advances, RAD51AP1 holds the potential to emerge as a novel target for precision therapy tailored to the molecular subtypes of endometrial carcinoma.

### 3.4 RAD51AP1 and Breast Cancer

Breast cancer is the most prevalent malignant tumor among women worldwide, with its incidence showing a consistent annual increase. It represents a significant threat to both the physical and mental health of women and has become a major public health concern [78]. Despite notable advancements in treatment modalities, including surgery, radiotherapy, chemotherapy, endocrine therapy, targeted therapy, and immunotherapy [79], recurrence and mortality rates remain high, particularly with a low 5‐year survival rate among patients with advanced breast cancer [80]. Notably, some patients with early‐stage breast cancer eventually develop recurrence and metastasis. Once distant metastasis occurs, treatment becomes significantly more challenging, leading to a substantial decline in patients' quality of life and survival time [81]. Therefore, identifying new molecular targets to enhance treatment efficacy and overcome drug resistance has become a primary focus of current research and an essential step toward addressing clinical challenges.

RAD51AP1 has been identified as exhibiting high expression in breast cancer. As early as 2007, Martin et al. [82] first used a method similar to bioinformatics analysis and found that RAD51AP1 was significantly overexpressed in breast cancer with breast cancer susceptibility gene 1 (*BRCA‐1*) deficiency. Pathania et al. [83] utilized the UCSC Cancer Genome Browser and GOBO gene enrichment analysis software to verify that RAD51AP1 expression in breast cancer was significantly higher than in normal breast tissue, particularly in triple‐negative basal‐like and Human Epidermal Growth Factor Receptor 2 (HER‐2) positive subtypes, where expression levels were even more elevated. Furthermore, high RAD51AP1 expression was strongly associated with poorer PFS in patients. Additionally, the team detected *RAD51AP1* mRNA expression in different breast cancer cell lines and found that its expression in triple‐negative breast cancer (TNBC) cell lines was significantly higher than in other subtypes. Le et al. [84] also indicated that RAD51AP1 was highly expressed in breast tissues of TNBC patients and correlated with poor prognosis. Bridges et al. [85] performed bioinformatics analysis by using the TCGA‐Breast invasive carcinoma (TCGA‐BRCA) database and found that breast cancer patients with high RAD51AP1 expression not only had worse OS but also lower recurrence‐free survival (RFS) post‐treatment. Thus, RAD51AP1 may serve as a crucial biomarker for guiding the prognosis of breast cancer patients.

RAD51AP1 may promote tumor proliferation and drug resistance by enhancing tumor stemness. Pathania et al. [83] demonstrated that the combined application of DNA methyltransferase (DNMT) and histone deacetylase (HDAC) inhibitors significantly reduced the number of breast cancer stem cells (CSCs) and resulted in a marked decrease in *RAD51AP1* transcription levels. This suggests that the suppression of RAD51AP1 may be a key mechanism through which these combined drugs inhibit tumor stemness. Further research [85] showed that in vitro knockdown of RAD51AP1 significantly reduced the expression of breast cancer‐related stemness markers (CD44, CD49f, NANOG, and KLF4), decreased the number of CSCs, and impaired clonogenic capacity. In vivo experiments using transgenic and nude mouse models also confirmed that RAD51AP1 enhances tumor proliferation and metastasis by promoting tumor stemness. Additionally, knockdown of RAD51AP1 in breast cancer cells significantly increased paclitaxel (PTX) and ionizing radiation‐induced apoptosis. Notably, significantly higher RAD51AP1 expression was observed in the PTX‐resistant CAL51‐PTX‐R cell line, and knocking down RAD51AP1 reversed drug resistance by reducing stemness. Huang et al. [86] further found that in TNBC tissues with high RAD51AP1 expression, not only was the cancer stem cell index (mRNAsi) elevated, but the expression of stemness‐related genes such as *KDM5B*, *BMI1*, *MYC*, and *EZH2* was also increased. These tissues also exhibited high resistance to multiple Mitogen‐Activated Protein Kinase Kinase (MEK)/extracellular regulated protein kinases (ERK) signaling pathway inhibitors (e.g., PD‐0325901, RDEA119, trametinib, and selumetinib). Subsequently, Liu et al. [87] performed RNA sequencing of breast cancer samples from the TCGA database and identified an enrichment of RAD51AP1‐DYRK4 fusion transcripts in Luminal B breast cancer. In vitro experiments confirmed that this fusion protein significantly enhances tumor cell invasiveness through a unique signaling network that activates the MEK/ERK pathway while inhibiting the PI3K/AKT pathway, reversing resistance to MEK inhibitors.

In summary, RAD51AP1's multifaceted role as a therapeutic target is highlighted by its subtype‐specific overexpression in high‐risk breast cancers (such as TNBC and HER2+), its regulation of stemness and therapy resistance, and its modulation of key oncogenic pathways (e.g., MEK/ERK and PI3K/AKT). Future research should focus on: (1) the differential mechanisms of RAD51AP1 and its fusion proteins across various breast cancer subtypes; (2) the synergistic interactions between RAD51AP1 and other important therapeutic targets (such as PARP). Clarifying these issues will provide a more precise theoretical basis for overcoming therapeutic resistance and optimizing combination therapies (e.g., targeting RAD51AP1 in combination with PARP inhibitors, chemotherapy, or radiotherapy), offering new perspectives for improving the survival and prognosis of patients with high‐risk breast cancer.

## 4 Conclusion and Perspectives

**FIGURE 2:**
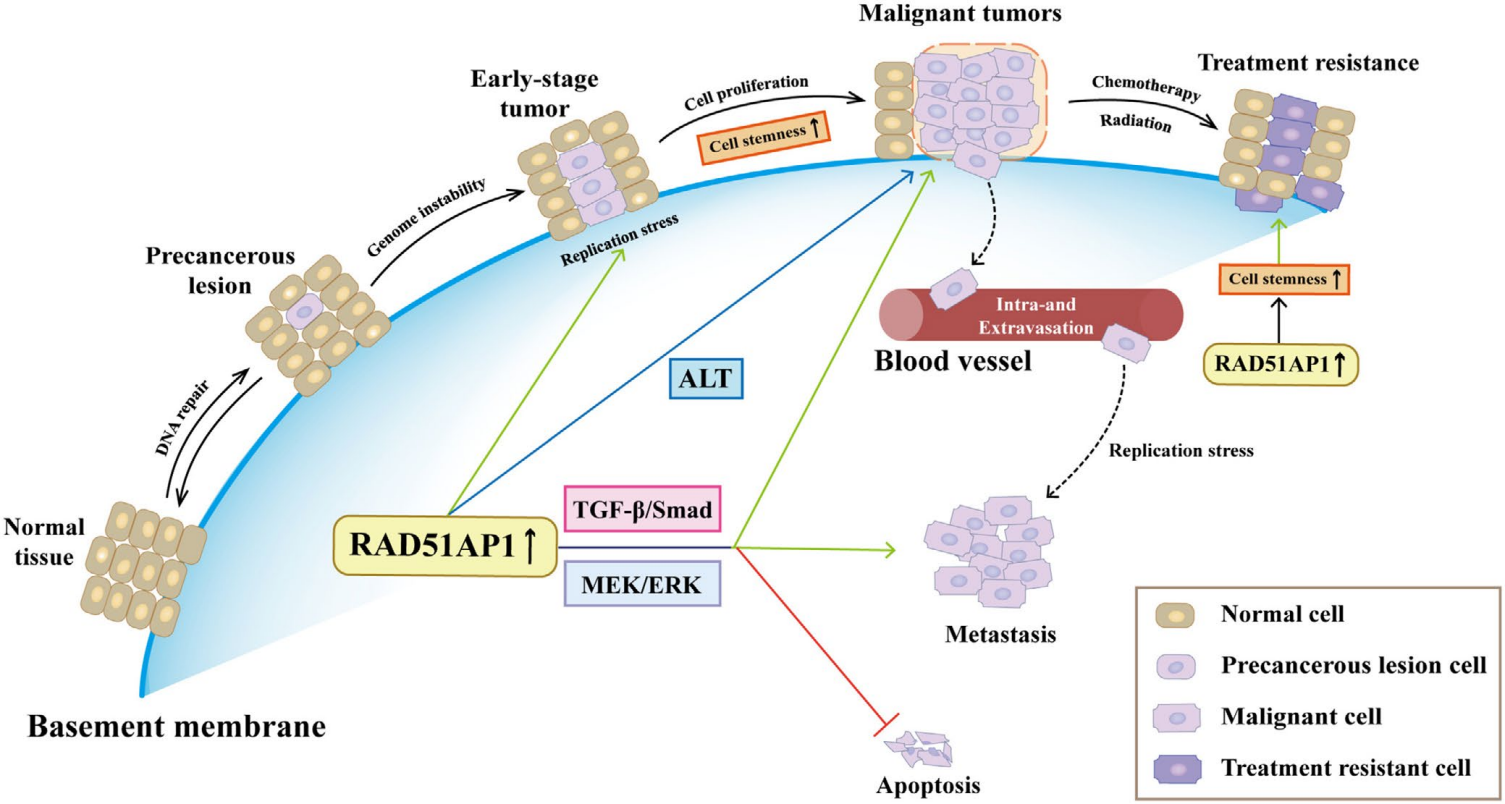
Model depicting elevated RAD51AP1 plays a critical role in tumor initiation, proliferation, metastasis, and treatment resistance when endogenous levels of DNA damage are high.

In conclusion, RAD51AP1 plays a pivotal role in HRR in normal cells while exhibiting extensive oncogenic effects (Figure 2). It is highly expressed in tissues of female reproductive system tumors, including ovarian, cervical, endometrial, and breast cancers, and is associated with poor prognosis (Table 1). RAD51AP1 promotes tumor proliferation, invasion, and inhibition of apoptosis, primarily through mechanisms such as enhancing tumor stemness and regulating key signaling pathways like TGF‐β/Smad and mTOR. Its ability to enhance tumor stemness is also a major mechanism by which it contributes to chemo‐radiotherapy resistance in female reproductive system tumors. Targeting RAD51AP1 and intervening in its upstream and downstream regulatory mechanisms could offer an important direction for addressing chemo‐radiotherapy resistance. However, several critical issues in this field remain to be explored. Future studies should focus on the following areas: (1) Clarifying the expression profile and regulatory mechanisms of RAD51AP1: Investigate the specific expression differences of RAD51AP1 across molecular subtypes of various malignant tumors (e.g., BRCA‐status subtypes in ovarian cancer, TNBC/Luminal subtypes in breast cancer) and their upstream regulatory mechanisms (e.g., transcriptional regulation, epigenetic modifications), to establish a foundation for precision treatment strategies based on molecular typing. (2) Decoding the signaling network and functional roles of RAD51AP1: Further explore the interaction network between RAD51AP1 and other key signaling pathways (e.g., metabolic pathways, immune regulatory pathways), clarify the molecular mechanisms through which it contributes to tumor metabolic reprogramming and immune microenvironment remodeling (e.g., polarization of tumor‐associated macrophages, inhibition of T cell infiltration), and reveal its broader role in tumor progression. (3) Verifying clinical translational value and advancing its application: Systematically validate the reliability of RAD51AP1 as an independent prognostic biomarker or as part of a prognostic model through multi‐center, large‐sample prospective clinical studies. Additionally, actively explore its potential in predicting treatment responsiveness (e.g., sensitivity to platinum‐based chemotherapy, PARP inhibitors, and radiotherapy) to accelerate its transformation into a clinical diagnostic marker and an aid for treatment decision‐making. RAD51AP1 is a critical molecule for understanding the mechanisms behind the onset and progression of female reproductive system malignancies and a promising target for therapeutic intervention. In‐depth future studies are expected to further elucidate its multifaceted biological functions and clinical value, providing crucial insights for developing novel targeted strategies and optimizing combination therapies (e.g., with PARP inhibitors, immunotherapy, or chemotherapy), ultimately opening new avenues for improving patient survival and prognosis.

**TABLE 1:** Research overview of RAD51AP1 in malignant tumors of the female reproductive system.

Tumor type	Expression status	Effects	Prognostic value (clinical significance)	Functional mechanism	References
Ovarian cancer	High expression in ovarian cancer tissues (particularly in primary and recurrent foci) and cells	High expression promotes proliferation, invasion, migration, and stemness maintenance effect of ovarian cancer cells; apoptosis inhibition; leading to cell cycle arrest in the G0/G1 phase; and mediates chemoresistance.	High expression is significantly correlating with poor prognosis (shortened OS and PFS), indicating an adverse prognosis.	Negatively regulated by hsa‐miR‐140‐3p and other molecules; regulates the mTOR, TGF‐β/Smad, and PI3K/AKT pathways; mediates HRR; targeting RAD51AP1 can reverse platinum resistance; selenides combined with chemotherapy can downregulate its expression and enhance sensitivity.	[53, 54, 55, 56, 57, 58, 59, 60, 61, 62]
Cervical cancer	Highly expressed in cervical cancer tissues and cell lines (e.g., SiHa, HeLa)	High expression promotes the proliferation and colony formation of cervical cancer cells; inhibits apoptosis; enhances DNA repair; and mediates radiotherapy resistance and MMC resistance.	High expression is associated with the proliferation and drug resistance of cervical cancer cells, but its correlation with prognosis remains unclear.	Small interfering RNA (siRNA)‐mediated knockdown can inhibit the proliferation of cervical cancer cells and induce apoptosis; transcriptionally activated by *E2F1* and epigenetically regulated by BRD4; the BRD4 inhibitor JQ1 can downregulate its expression and enhance radiotherapy sensitivity; regulates the TGF‐β/Smad pathway; enhances HRR capacity.	[69, 70, 71]
Endometrial cancer	Highly expressed in endometrial cancer tissues and cell lines	Promotes the proliferation and survival of endometrial cancer cells; inhibits preadipocyte differentiation; enhances stemness; mediates 5‐FU resistance; associated with fatty acid metabolic reprogramming.	High expression is associated with decreased survival rate.	Promotes the occurrence and development of endometrial cancer by enhancing tumor stemness; targeting RAD51AP1 or its upstream hsa‐miR‐383‐5p may reverse chemotherapy resistance; mediates chemotherapy resistance through the fatty acid metabolism pathway.	[20]、 [76, 77]
Breast cancer	Highly expressed in breast cancer tissues (especially TNBC and HER2+ subtypes) and cell lines	Promotes the proliferation, metastasis, colony formation, and stemness maintenance of breast cancer cells; inhibits therapy‐induced apoptosis; mediates paclitaxel resistance and radiotherapy resistance; confers resistance to multiple MEK inhibitors.	High expression is significantly associated with poor PFS, RFS, and OS; it is a potential prognostic biomarker.	Combined use of DNMT/HDAC inhibitors can reduce its expression; regulates stemness markers such as CD44/NANOG/KLF4; targeting RAD51AP1 can enhance sensitivity to paclitaxel and radiotherapy; affects the MEK/ERK and PI3K/AKT pathways; the RAD51AP1‐DYRK4 fusion exists and activates MEK/ERK.	[82, 83, 84, 85, 86, 87]

## Author Contributions

This work complies with the ICMJE criteria for authorship, which require significant contributions to conception/design, data acquisition/analysis, manuscript drafting/revision, and final approval. The authors' contributions are specified using the CRediT taxonomy as follows: **Chengguo Zhang:** conceptualization (literature synthesis and review framework), investigation (data collection and analysis), writing – original draft (manuscript preparation). **Xiaoyang Liu:** data curation (literature organization and validation), writing – review and editing. **Zizhang Li:** formal analysis (pathway mechanism interpretation). **Jiayao Han:** methodology (review strategy development), validation (data consistency verification). **Jun Liang:** supervision (project oversight), Funding acquisition (grant administration), writing – review and editing (critical intellectual content). **Nan Zhou:** conceptualization: led the overall research conception of the review and the design of its core framework, supervision: comprehensively supervised the entire review research process and guided the core work of all authors, writing – review and editing: Conducted the final critical review of the full text. All authors agree to be accountable for all aspects of the work and confirm compliance with the ICMJE guidelines.

## Conflicts of Interest

The authors declare no conflicts of interest.

## Supporting information


**Data S1:** fsb271155‐sup‐0001‐TableS1.docx.

## Data Availability

No new datasets were generated or analyzed in this review article. The study synthesizes existing preclinical and clinical data from previously published research, which are cited in the references. Therefore, data sharing is not applicable to this manuscript.
