# Supplementary material for: Research Progress of RAD51AP1 in Malignant Tumors of the Female Reproductive System
Source: FASEB J. 2025 Oct 19;39(20):e71155. doi: 10.1096/fj.202502048R (PMC12535699; doi:10.1096/fj.202502048R)
Supplement: Supplementary file 1 — Data S1: fsb271155‐sup‐0001‐TableS1.docx. [file FSB2-39-e71155-s001.docx]

| 3'UTRs | 3' untranslated regions |
| --- | --- |
| 5-FU | 5-fluorouracil |
| AKT | protein kinase B |
| ALT | alternative lengthening of telomeres |
| *ATM* | ataxia telangiectasia-mutated gene |
| ATR | ataxia telangiectasia and Rad3-related protein |
| BITS | break-induced telomere DNA synthesis |
| *BRCA-1* | breast cancer susceptibility gene 1 |
| *BRCA2* | breast cancer susceptibility gene 2 |
| BRD4 | bromodomain-containing protein 4 |
| CDK1 | cyclin-dependent kinase 1 |
| CDX | cell-line derived xenograft |
| CESC | Cervical squamous cell carcinoma and endocervical adenocarcinoma |
| cryo-EM | Cryo-electron microscopy |
| CSCs | cancer stem cells |
| DDR | DNA damage repair |
| D-loop | displacement loop |
| DNMT | DNA methyltransferase |
| DSB | double-strand break |
| ERK | extracellular regulated protein kinases |
| G4s | G-quadruplexes |
| GEO | Gene Expression Omnibus |
| GEO | Gene Expression Omnibus |
| GTEx | Genotype-Tissue Expression |
| HDAC | histone deacetylase |
| HER-2 | Human Epidermal Growth Factor Receptor 2 |
| HPV | Human Papillomavirus |
| HRR | Homologous recombination repair |
| IC50 | median inhibition concentration |
| IHC | immunohistochemical |
| KLF4 | Kruppel-like factor 4 |
| MEK | Mitogen-Activated Protein Kinase Kinase |
| miRNAs | microRNAs |
| MMC | mitomycin C |
| MRE11 | meiotic recombination 11 |
| mRNAsi | mRNA Stemness Index |
| mTOR | proteins associated with the mammalian target of rapamycin |
| OCT4 | octamer-binding transcription factor 4 |
| OS | overall survival |
| OV | ovarian serous cystadenocarcinoma |
| *PALB2* | partner and localizer of *BRCA2* |
| PARP | poly-ADP-ribose polymerase |
| PCNA | proliferating cell nuclear antigen |
| PFS | progression-free survival |
| PI3K | phosphatidylinositol 3-kinase |
| Polη | polymerase η |
| PTX | paclitaxel |
| RAD51AP1 | RAD51-associated protein 1 |
| RFS | recurrence-free survival |
| RPA | Replication Protein A |
| SOX2 | sex-determining region Y-box2 |
| ssDNA | single-stranded DNA |
| TCGA | The Cancer Genome Atlas |
| Tensor GSVD | Tensor Generalized Singular Value Decomposition |
| TGF-β | transforming growth factor-β |
| TNBC | triple-negative breast cancer |
| UCEC | Uterine Corpus Endometrial Carcinoma |
